# Anti-inflammatory effects of intravenous methotrexate associated with lipid nanoemulsions on antigen-induced arthritis

**DOI:** 10.6061/clinics/2016(01)09

**Published:** 2016-01

**Authors:** Suzana B V Mello, Elaine R Tavares, Maria Carolina Guido, Eloisa Bonfá, Raul C Maranhão

**Affiliations:** Instituto do Coração (InCor), Faculdade de Medicina da Universidade de São Paulo; I Divisão de Reumatologia,; II Laboratório de Metabolismo de Lipídios, São Paulo/, SP, Brazil; III Universidade de São Paulo, Faculdade de Ciências Farmacêuticas, São Paulo/, SP, Brazil

**Keywords:** Nanoparticles, Methotrexate, Rheumatoid Arthritis, Experimental Arthritis, Solid Lipid Particles

## Abstract

**OBJECTIVE:**

To test the hypothesis that intravenous use of methotrexate associated with lipid nanoemulsions can achieve superior anti-inflammatory effects in the joints of rabbits with antigen-induced arthritis compared with commercial methotrexate.

**METHODS:**

Arthritis was induced in New Zealand rabbits sensitized with methylated bovine serum albumin and subsequently intra-articularly injected with the antigen. A nanoemulsion of methotrexate labeled with ^3^H-cholesteryl ether (4 mg/kg methotrexate) was then intravenously injected into four rabbits to determine the plasma decaying curves and the biodistribution of the methotrexate nanoemulsion by radioactive counting. Additionally, the pharmacokinetics of the methotrexate nanoemulsion were determined by high-pressure liquid chromatography. Twenty-four hours after arthritis induction, the animals were allocated into three groups, with intravenous injection with saline solution (n=9), methotrexate nanoemulsion (0.5 µmol/kg methotrexate, n=7), or commercial methotrexate (0.5 µmol/kg, n=4). The rabbits were sacrificed 24 h afterward. Synovial fluid was then collected for protein leakage and cell content analyses and synovial membranes were collected for histopathological analysis.

**RESULTS:**

The methotrexate nanoemulsion was taken up mainly by the liver and the uptake by arthritic joints was two-fold greater than that by control joints. The methotrexate nanoemulsion treatment reduced leukocyte influx into the synovial fluid by nearly 65%; in particular, mononuclear and polymorphonuclear cells were reduced by 47 and 72%, respectively. In contrast, cell influx was unaffected following treatment with commercial methotrexate. Protein leakage into the arthritic knees of the rabbits was also more limited following methotrexate nanoemulsion treatment than following commercial methotrexate treatment.

**CONCLUSIONS:**

The intravenous methotrexate nanoemulsion showed anti-inflammatory effects on the synovia of arthritic joints that were clearly superior to the effects of a commercial methotrexate preparation. This result is conceivably due to greater methotrexate uptake by the joints when the drug is associated with a nanoemulsion.

## INTRODUCTION

In a recent study, we showed that a preparation consisting of a methotrexate (MTX) derivative, namely, didodecyl MTX, associated with a lipid nanoemulsion (LDE) had the ability to markedly reduce inflammation when injected into rabbits with antigen-induced arthritis (AIA) via the intra-articular route. This effect was not attained when a commercial MTX preparation at the same dose was intra-articularly injected into rabbits 1. In fact, it is well known that MTX is not effective when administered directly into a joint.

As shown by Maranhão et al. 2,3, the targeting capabilities of the LDE system are attained by constructing LDEs with a composition and structure that resemble those of low-density lipoprotein (LDL), but without proteins. LDEs have affinity for the different exchangeable apolipoproteins (apo) contained in the plasma. One of the acquired apo molecules, or apo E, is recognized by the LDL receptor and is thereby internalized into cells via a receptor-mediated pathway. In cancer, atherosclerosis, organ graft rejection, arthritis, and other diseases that include proliferative and inflammatory processes, the lipoprotein receptors are up-regulated, so LDEs and drugs associated with the nanoemulsions can be concentrated at targeted sites. The reduction in the toxicity of chemotherapeutic agents is remarkable, as shown in experimental animals 4-6 and in clinical trials enrolling patients with advanced cancer 7-9. Moreover, the pharmacological action of the agents can be increased using this drug targeting system 10,11.

MTX used in monotherapy or in combination with other drugs is the drug of choice for the treatment of rheumatoid arthritis (RA). RA is an autoimmune disease and one of the principal causes of joint pain. RA is specifically characterized by symmetric, peripheral polyarthritis. Inflammation of the cells lining the synovium produces progressive erosion of the synovial joints and often results in joint damage and disability, along with several other systemic manifestations, such as an increased incidence of ischemic heart disease.

MTX is a folic acid antagonist that has both anti-proliferative and immunosuppressive actions. This drug also has an extensive toxicity range and, in addition to its relatively high toxicity, the variability and unpredictability of its pharmacological action are also drawbacks to the use of MTX in RA treatment. Due to poor response, high toxicity, or both, approximately 26% of patients are expected to discontinue MTX treatment.

MTX is hydrophilic and cell uptake of this compound, which depends on the folate receptor pathway, is poor, constituting a limiting factor for the therapeutic effectiveness of the drug and favoring the development of resistance to treatment. Association with an LDE increases the uptake of MTX by cultured neoplastic cells (K562 and HL60 cell lineages) up to ninety-fold as the drug is internalized via LDL-receptor-mediated endocytic pathways, instead of the folate receptors 12. When injected into the inflamed joints of rabbits with AIA, the uptake of a radioactive LDE by arthritic joints is 2.5-fold greater than uptake by normal joints. To achieve an optimum yield for MTX incorporation into an LDE and to improve the stability of the preparation of MTX associated with an LDE (LDE-MTX), MTX was previously esterified with dodecyl bromide.

In view of the positive outcome of the experiments with intra-articular LDE-MTX, experiments were designed to test the hypothesis that the systemic use of LDE-MTX can also achieve superior anti-inflammatory effects in comparison with commercial MTX. The pharmacokinetics and biodistribution of the novel formulation were determined in control rabbits and the anti-inflammatory effects were investigated in an antigen-induced RA rabbit model.

## METHODS

The experiments were approved by the Animal Ethics Committee of the Brazilian College of Experimental Animals (CAPPesq 1093/07). Preparation of LDE-MTX was performed as described in our previous study 1.

The pharmacokinetics of LDE-MTX were determined using high-pressure liquid chromatography (HPLC) equipment (Model SPD-10AV, Shimadzu Corporation, Kyoto, Japan using a UV detector at 300 nm). Chromatographic separation was achieved with a ShimPack C_18_ 2 (5μm, 15 cm x 6 mm) analytical column (Phenomenex, Torrance, CA) and protected by a Luna C_18_ 2 guard cartridge. The mobile phase was methanol at a flow rate of 1 mL/min for MTX. Stock solutions of MTX were prepared at 0.1 mg/mL in methanol and stored at 4°C. A stock solution of N-oleyl daunorubicin, used as an internal standard (IS), was diluted in methanol and stored at 4°C. Thawed blank plasma was spiked with the 0.1 mg/mL MTX stock solution to prepare a 5000 ng/mL primary plasma stock, which was diluted further with blank plasma to prepare plasma standards of 78.125, 156.25, 312.5, 625, 1250, 2500 and 5000 ng/mL. To each tube, 50 μL (5000 ng) of the IS was added and evaporated under nitrogen flow, after which 300 μL of plasma standard and 600 μL of ethyl acetoacetate:isopropyl alcohol (1:1) were added. The tubes were vortexed for approximately 1 min and then centrifuged at 3500 rpm for 15 min, after which 500 μL of supernatant was transferred into a clean tube and evaporated at 36°C under nitrogen. The residue was reconstituted with 50 μL of methanol and 20 μL was injected onto the HPLC system to set up the calibration curve.

Extraction of the MTX by precipitation with ethyl acetoacetate:isopropyl alcohol (1:1) was used for rabbit plasma sample preparation. In particular, rabbit plasma standards or samples (300 μL) containing MTX were aliquoted into 5 mL tubes, spiked with 50 μL (5000 ng) of the IS and then mixed with ethyl acetoacetate:isopropyl alcohol (1:1). The tubes were vortexed for approximately 1 min and then centrifuged at 3500 rpm for 15 min, after which 500 μL of supernatant was transferred into a clean tube and evaporated at 36°C under nitrogen. The residue was reconstituted with 50 μL of the mobile phase (methanol) and 20 μL was injected onto the HPLC system at 1 mL/min. All calculations were performed using the corrected peak area (peak area/migration time). Calibration graphs were calculated by analyzing seven different standard solutions of MTX using the IS method and weighted linear regression (1/x).

The pharmacokinetics of MTX were determined in control rabbits weighing approximately 3 kg. For this purpose, LDE-MTX (2 mL total volume; 60 mg total lipids and 12 mg drug) was injected as a single bolus into the marginal ear vein of four rabbits for determination of the plasma decaying curves. Blood samples were collected at pre-established intervals over a 24 h period (5 and 15 min and 1, 2, 4, 8 and 24 h). The plasma was separated by a 15 min centrifugation (3,000 g) and the concentration of MTX was then determined by HPLC. The pharmacokinetic parameters of MTX were calculated using a multicompartmental model via computer software (PK Solutions, Ashland, OH).

A 100 μL volume of LDE-MTX labeled with ^14^C-cholesteryl ether (Perkin Elmer, Boston, MA) was intravenously injected into the ear vein of 3 rabbits. The animals were kept in individual cages, and 24 h after injection, they were sacrificed. Fragments of liver, spleen, lung, heart, kidney, muscle, pancreas and synovial tissue were excised and kept in cold saline solution prior to lipid extraction with chloroform:methanol (2:1 v/v) 13. After lipid extraction, the solvent was evaporated under nitrogen flow and resuspended with 500 µL of chloroform:methanol (2:1 v/v) and half the suspension was placed into vials with 5 mL of scintillation solution (Packard BioScience, Groeningen, Netherlands). The radioactivity was then measured with a Packard 1600 TR liquid scintillation spectrometer (Palo Alto, CA).

Arthritis induction in New Zealand White rabbits was performed as described in our previous study 1. Twenty-four hours after arthritis induction, the animals were allocated into three groups: one group was intravenously injected with saline solution (300 µL, n=9); the second group, with LDE-MTX (0.5 µmol/kg MTX, n=7); and the third group, with commercial MTX (Miantrex®, Pfizer, New York, USA) (0.5 µmol/kg, n=4). All preparations were injected in a single dose.

The animals were sacrificed 48 h after arthritis induction. Immediately after sacrifice, 2 mL of saline containing EDTA (1 mg/mL) was injected into each knee joint. Synovial fluid was collected with a needle, and the joint was then opened to recover the remaining fluid. The collected synovial fluid was diluted, and the total cell count was determined by light microscopy. A differential cell count was also obtained using smears prepared from cell pellets and stained with Giemsa.

To assess the vascular permeability, before the induction of arthritis, the animals had been intravenously injected with 20 mg/kg Evans Blue in 2.5% saline solution. This dye binds to plasma proteins, tagging macromolecule that cross the endothelial barrier only when tissues are inflamed. The joint fluid was centrifuged and the density was assessed using a colorimetric method at 630 nm. The dye concentration in the joint was estimated from a standard curve of Evans Blue serial dilutions in saline solution. The results are expressed as µg of protein/mL of synovial fluid.

## RESULTS AND DISCUSSION

**Figure 1-:**
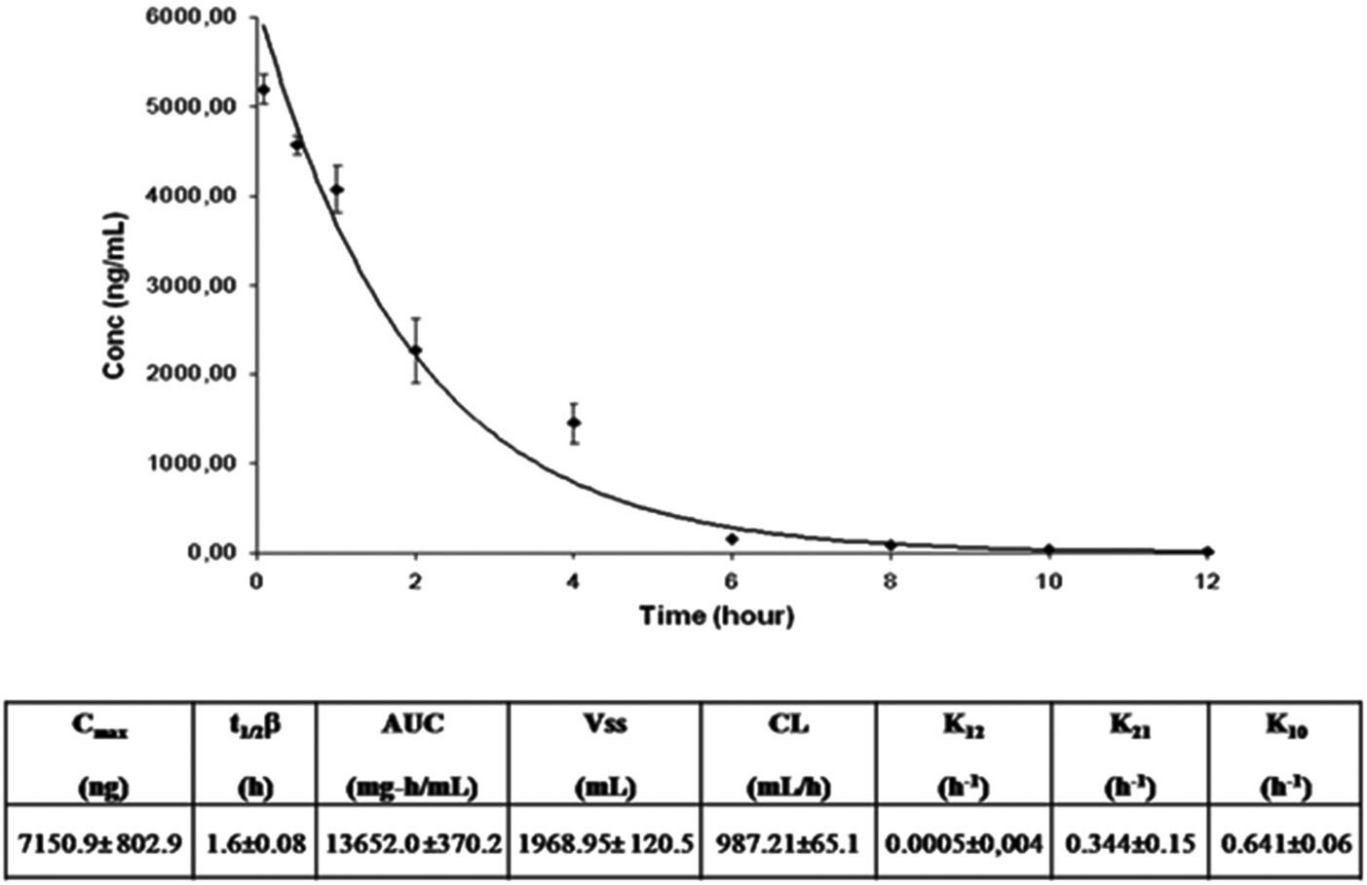
Decay curve of didodecyl MTX associated with an LDE after a bolus injection of 3.6 mg/kg drug and pharmacokinetic parameters obtained from the curve. The concentration of MTX in the plasma was determined by HPLC. The analysis was performed using a two-compartment open model. t1/2β: elimination half-life; AUC: area under the plasma concentration-time curve; Vss: volume of distribution at steady state; CL: total body clearance. The results are expressed as the mean ± SE of data obtained from 4 rabbits.

The standard and calibration curve equations showed that the concentrations of MTX had a good linear correlation (n=7, r^2^=0.999) for the standard solution of the drug in serum samples in the concentration range of 78.125 to 5000 ng/mL. Figure 1 shows the plasma kinetics of MTX associated with the nanoemulsion after a bolus intravenous injection into the rabbits, as followed over 24 h and determined by HPLC. The kinetic parameters derived from the curve are displayed in Figure 1.

**Figure 2-:**
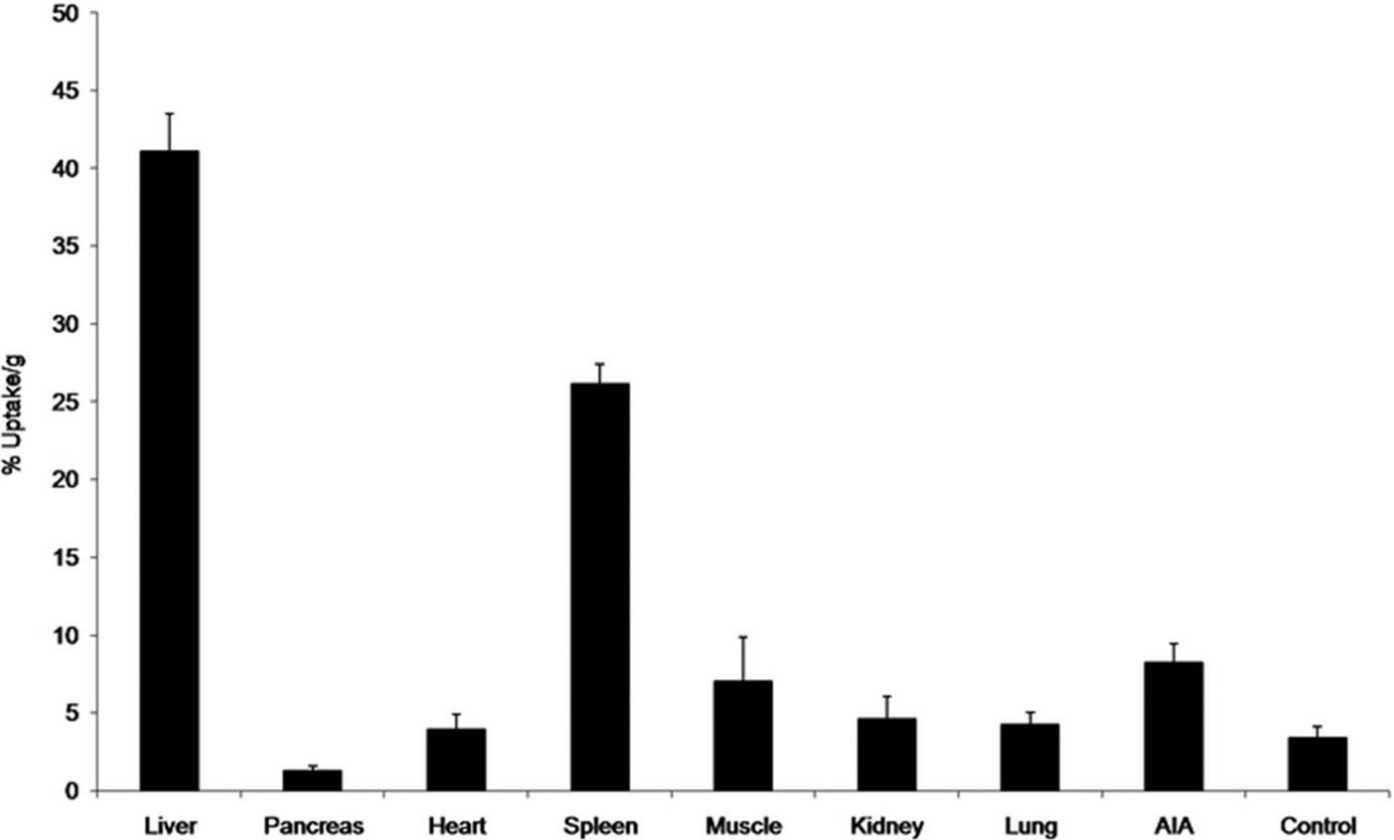
Tissue uptake of LDE labeled with ^3^H-cholesteryl oleyl ether. The tissue samples were excised for radioactivity counting 24 h after intravenous injection of the labeled LDE into the rabbits with AIA. Contralateral synovia without arthritis induction were used as controls.

Regarding the biodistribution of the LDE^ 3^H-cholesterol oleate ether in rabbits with AIA, the liver was the main tissue with uptake, followed by the spleen. Uptake by arthritic joints was two-fold greater than uptake in control saline-injected joints, representing the tissue with the third highest uptake (Figure 2).

**Figure 3-:**
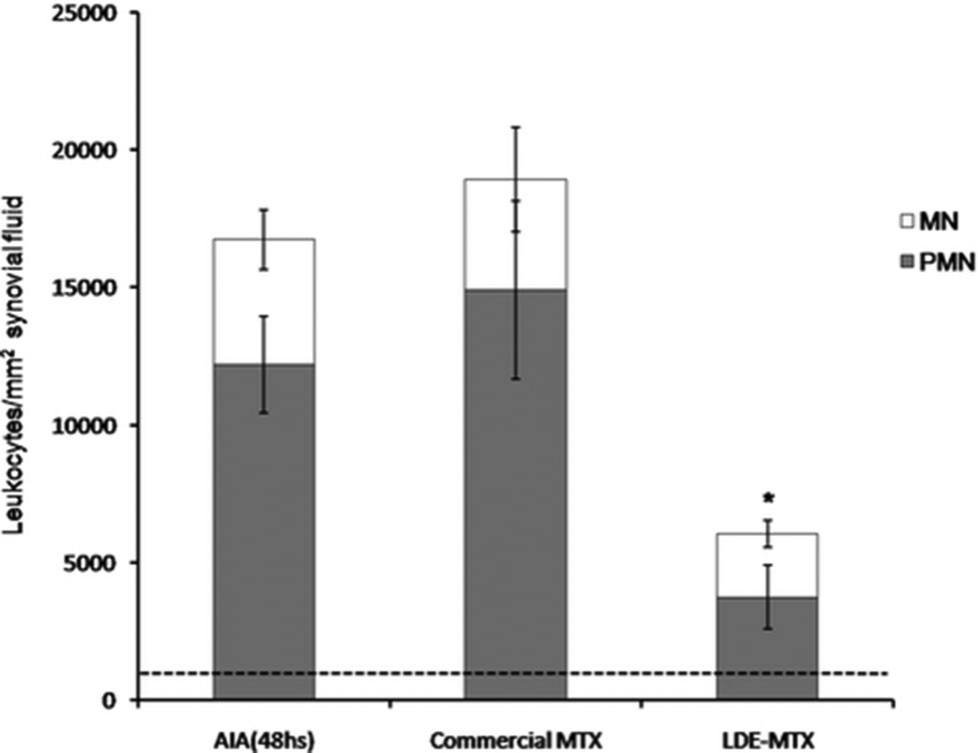
Leukocyte count (monocytes: white column; polymorphonuclear cells: gray column) in the synovial fluid of rabbits 48 h after AIA induction. The animals received commercial MTX (0.5 µmol/kg), LDE-MTX (0.5 µmol/kg) or saline solution intravenously 24 h after AIA induction. * p<0.05 compared with the control saline-treated rabbits with AIA. The results are expressed as the mean ± SEM. The data have been subjected to repeated analysis of variance. Post-analysis was conducted using Newman-Keuls multiple comparison tests. In all analyses, p<0.05 was considered statistically significant.

**Figure 4-:**
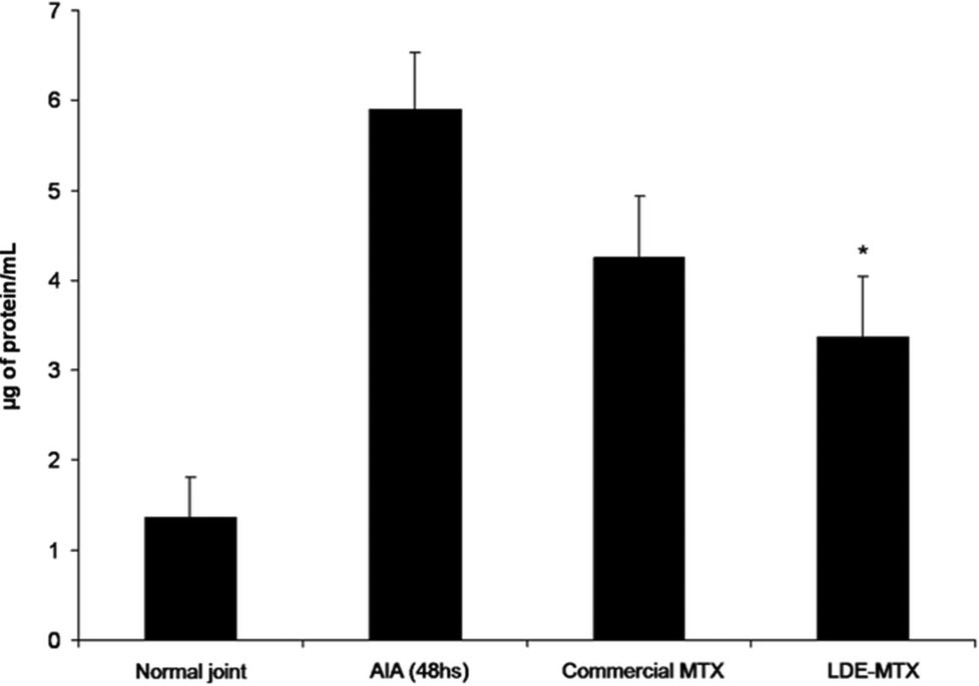
Vascular permeability, as assessed by the Evans Blue method, in joint fluid collected from the joints of rabbits 48 h after AIA induction. The animals received intravenous commercial MTX (0.5 µmol/kg), LDE-MTX (0.5 µmol/kg) or saline solution 24 h after the induction of arthritis. * *p*<0.05 compared with the control saline-treated rabbits with AIA. The results are expressed as the mean ± SEM. The data have been subjected to repeated analysis of variance. Post-analysis was conducted using Newman-Keuls multiple comparison tests. In all analyses, *p*<0.05 was considered statistically significant.

Figure 3 shows the total and differential leukocyte counts in the synovial fluid of the arthritic joints of the rabbits with AIA treated with saline, commercial MTX or LDE-MTX. Commercial MTX was ineffective in reducing the leukocyte influx into the articular cavity. In contrast, the LDE-MTX preparation reduced the leukocyte influx by nearly 65%; specifically, LDE-MTX treatment reduced the mononuclear (47%) and polymorphonuclear (72%) cell counts. The latter are the predominant cell lineage in this phase of the inflammatory process. The superior anti-inflammatory efficacy of LDE-MTX was also shown by the data on the protein leakage into the arthritic knees of the rabbits with AIA, which was more limited in this treatment group compared with both controls and commercial MTX-treated rabbits with AIA (Figure 4).

Several drug delivery systems have been proposed to improve the pharmacological index of MTX. Those systems include liposomes, dendrimers, human serum albumin, solid lipid nanoparticles, polymeric nanoparticles and micelles, carbon nanotubes, and magnetic and gold nanoparticles. At this stage, it is difficult to evaluate the advantages of one system over the others because most of studies have been pre-clinical testing reports. With respect to the MTX preparations tested in subjects, namely, liposomes and human serum albumin, there is still a lack of clinical evidence to support their overall superiority 14,15. Being a low-cost oral medication with widespread and long-standing clinical use in RA and other rheumatic diseases, it is unlikely that commercial MTX could be replaced by parenteral drug delivery systems in the setting of chronic treatment of those diseases. However, periods of treatment with drug delivery systems that can increase the anti-inflammatory action of MTX could reverse the stages of disease aggravation demanding prompt intervention, especially when orally administered medications are not effective.

The potential advantages of LDE-MTX are its lack of immunogenicity and toxicity related to nanotechnological products, which have become major issues in the field. The fact that the LDE constituent materials are only lipids, which are present in the organism and are furnished by the chemical industry; the low cost; and the ease of large-scale production are also chief advantages.

It is noteworthy that the anti-inflammatory effects of intravenous LDE-MTX injection, as illustrated in Figures 3 and 4, closely resemble those obtained following injection by the intra-articular route 1. The overwhelming superiority of the LDL-receptor-mediated endocytic pathway over the folate receptor in promoting cell uptake of MTX 16 may have been responsible for the marked improvement of the drug's anti-inflammatory properties when the LDE was used as a carrier. In commercial MTX treatment, low cell uptake via the folate receptor can conceivably facilitate drug resistance. In view of the current results in the AIA rabbit model, the possibility exists that use of an LDE as an MTX carrier may also have the potential to disrupt drug resistance mechanisms to allow the continuation of MTX treatment.

## AUTHORS CONTRIBUTION

Melo SB was responsible for the design of the study and the data analysis. Tavares ER was responsible for the technical execution and the manuscript writing. Guido MC helped with the manuscript writing. Bonfá E and Maranhão RC participated in the study design and the manuscript writing and provided financial support.
